# Efficacy and safety of a sustained-release formulation of ivermectin (FILAPREV®) in preventing heartworm infection (*Dirofilaria immitis*) in dogs in two endemic areas of Italy

**DOI:** 10.1186/s13071-026-07419-9

**Published:** 2026-05-05

**Authors:** Marco Genchi, Luigi Venco, Marta Fozzer, Alessia Crippa, Claudio N. Rossi, Laura Kramer, Alice Vismarra

**Affiliations:** 1 Department of Veterinary Medicine Sciences, University of Parma, Parma, Italy; 2 Department Veterinary Sciences, University of Turin, Turin, Italy; 3 Ceva Santé Animale, 8 rue de Logrono, 33500 Libourne, France

**Keywords:** *Dirofilaria immitis*, Ivermectin, Implant, Slow release, Moxidectin, Heartworm prevention

## Abstract

**Background:**

Macrocyclic lactones are the only drug class currently licensed for heartworm disease prophylaxis. Macrocyclic lactones kill third- and fourth-stage larvae of the parasitic nematode *Dirofilaria immitis*, thus preventing the development of adult worms in dogs, which are responsible for heartworm disease, a potentially life-threatening condition. The objective of the present study was to assess the efficacy of a subcutaneous implant formulation containing ivermectin (FILAPREV®; Ceva Santé Animale, Libourne, France) compared to extended-release moxidectin (Guardian™ SR; Elanco, Indianapolis, IN, USA) to protect dogs against *D. immitis* infection during the entire transmission-risk season in Italy, starting in April and ending in November.

**Methods:**

A total of 114 healthy client-owned dogs were enrolled at two investigation sites located in two different regions of Italy known to be endemic for *D. immitis* (Lombardy and Veneto). In the spring of 2017, approximately half of the dogs from each site randomly received a single dose of FILAPREV® (ivermectin, 0.13 mg/kg as minimum standard dose); the remaining dogs were treated with a single dose of Guardian™ SR (moxidectin, 0.17 mg/kg). Antigen and Knott’s tests were performed before treatment, and subsequently at 1 week and 6, 8 and 12 months post-treatment. Clinical examination, thoracic radiography and echocardiography were conducted at the same time points.

**Results:**

Of the 114 dogs enrolled in the study, 112 completed the entire study period. No adverse reactions were observed, and all dogs enrolled in the study remained heartworm-negative throughout the entire study period.

**Conclusions:**

A single subcutaneous administration of FILAPREV®, an implant containing ivermectin at the recommended dosage, prevents heartworm disease caused by *D. immitis* in dogs for at least 8 months, covering the full seasonal period of heartworm transmission in hyperendemic regions.

**Graphical abstract:**

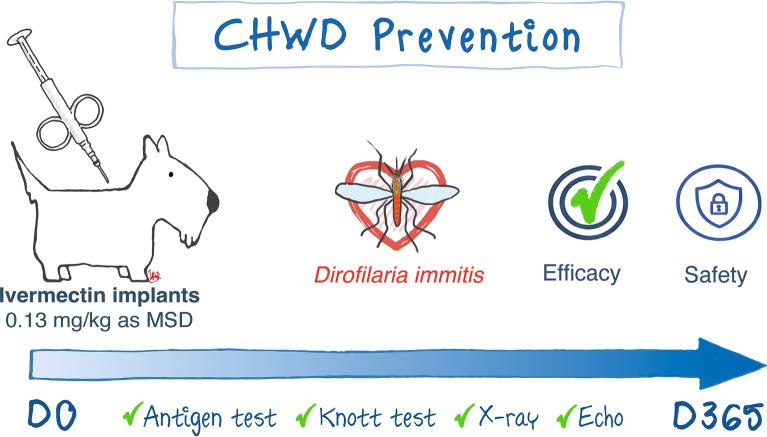

## Background

*Dirofilaria immitis,* the causative agent of canine heartworm disease (CHWD), is a mosquito-borne filarial parasite that is endemic in several European countries [1]. Heartworm disease in dogs is a chronic and progressive condition that can potentially lead to life-threatening pulmonary thromboembolism and right-side congestive heart failure if left untreated [2].

In Italy, one of the areas of highest prevalence for CHWD is along the Po River Valley in the northern region of the country [3], and a recent study reported that approximately 16% of dogs in this area tested positive for *D. immitis* [4]. In addition, reports of *D. immitis* infections in central and southern regions of Italy have been increasing in recent years [5]. The spread of heartworm disease is driven by several factors, including an increase in the movement of dogs, the introduction of competent mosquito vectors and a lack of awareness among veterinary practitioners regarding the need to administer preventive drug products containing a macrocyclic lactone (ML) [5].

The prevention of CHWD is based on the larvicidal activity of macrocyclic lactones (MLs), which eliminate migrating third-stage (L3) and fourth-stage larvae (L4) before they can reach maturity. These drugs are administered during the transmission season which, in northern Italy, is standardly from April to November [6]. Commonly used MLs for preventing CHWD include oral and or topically administered ivermectin, milbemycin oxime and moxidectin. In several countries, including Italy, moxidectin is also available as an extended-release, long-acting injectable formulation [7].

The objective of the present study was to assess the ability of subcutaneous implants containing ivermectin (FILAPREV®; Ceva Santé Animale, Libourne, France) to protect dogs against *D. immitis* infection throughout the full seasonal period of risk in Italy.

## Methods

### Animals

This randomized, double-blinded study was carried out from April 2017 to May 2018, in accordance with VICH (International Cooperation on Harmonization of Technical Requirements for Registration of Veterinary Medicinal Products) Guideline 9 (GL9) on Good Clinical Practice.

Healthy client-owned dogs were enrolled from two investigation sites located in two different regions of Italy known to be endemic for *D. immitis* (Veneto and Lombardy regions). The inclusion criteria are reported in Table 1 and included body weight ≥ 2 kg and < 60 kg; age ≥ 12 weeks; negative test result for adult heartworm antigen and circulating microfilariae (mf); normal cardiac and pulmonary functions. Any dogs with post-inclusion removal criteria were withdrawn from the study (Table 1).

**Table 1:** Inclusion and post-inclusion removal criteria

Criteria category	Full description of criteria
Inclusion criteria	Dogs of any gender and breed fulfilling all of the following inclusion criteria: - The owner had signed the informed consent form - Body weight ≥ 2 kg and < 60 kg - At least 12 weeks of age - Negative for adult heartworm antigen with onsite antigen test (SNAP 4DX Plus®) and negative for laboratory antigen (PetChek®) and for microfilariae at day 8 and day 0; - Lived in, and whose owner intended to remain in the area of the selected study site (Veneto or Lombardy) - Clinically healthy, with normal cardiac and pulmonary functions
Post-inclusion removal criteria	Dogs presenting at least one of the following conditions after inclusion were excluded from the study during the follow-up: - Leaving the study regions (Veneto and Lombardy) for > 3 weeks during the study - Testing positive for microfilariae, adult antigen and/or after X-ray and/or echocardiography findings suggestive of heartworm infection at the day 0, day 8 and/or day 120 visits (post-inclusion detection of a pre-patent infection) - Testing positive for microfilariae or adult heartworm antigen and/or after X-ray and/or echocardiography findings suggestive of heartworm infection at the day 245 visit - Receiving any parasiticide containing macrocyclic lactones, heartworm adulticide (melarsomine), pyrethroid-based repellents and/or tetracyclines during the study period - Experiencing an adverse event requiring interruption of treatment (in such cases, the drug dispenser would be contacted for unblinding and potential implant removal for dogs treated with FILAPREV®) - Experiencing death or euthanasia

Half of the dogs included in the study were crossbreed dogs (52.6%). The most common pure breeds included Dachshund, Italian Bloodhound (6.1%), Labrador Retriever (5.3%), Jack Russel and Yorkshire Terriers (4.4%) and Shih Tzu (2.6%). Forty-six (40.4%) of the dogs were male and 68 (59.6%) were female; 47 (41.2%) were intact and 67 (58.8%) were neutered. The mean age was 6.3 (range 0.5–17.3) years. The majority (102/114) were adult dogs (age ≥ 18 months), while the remaining 12 dogs were considered to be still growing (age < 18 months). The mean body weight was 15.7 (range 2.9–51.0) kg.

Fifty-nine dogs were recruited from the Veneto region and 55 dogs were recruited from the Lombardy region. At each site, dogs were randomly allocated into two groups: 58 dogs (30 from Veneto and 28 from Lombardy) received a single dose of FILAPREV® (ivermectin, 0.13 mg/kg as minimum standard dose [MSD]). FILAPREV® is a ready-to-use device which consists of a needle pre-filled with one or more implant subunit(s) based on body weight (BW) range and a reusable syringe. Each implant contains 2 mg of ivermectin and 6 mg of polylactid. FILAPREV® 1was administered by one subcutaneous injection between the shoulder blades. The remaining 56 dogs (29 from Veneto and 27 from Lombardy) received a single dose of Guardian™ SR (moxidectin, 0.17 mg/kg) (Elanco, Indianapolis, IN, USA)^2^. Guardian™ SR was administered by one subcutaneous injection under the loose skin on the dorsum or at the side of the neck, anterior to the scapula. Before injections, the skin area was disinfected with 2% chlorhexidine. The study design is reported in Table 2.

**Table 2:** Study design

Investigators	Activity	Day^a^						
		D-8	D0	D8	D30	D120	D245	D365
Clinical investigator	Clinical examination	X	X	X	X	X	X	X
	Body weight	X	X	X	X	X	X	X
	Thoracic radiography	X^b^	X^b^	X^b^		X^b^	X^b^	X^b^
	Echocardiography	X^b^	X^b^	X^b^		X^b^	X^b^	X^b^
	Onsite antigen test (SNAP 4DX Plus®)	X	X	X		X	X	X
	Lab antigen test (PetChek®) Knott’s test	X	X	X		X	X	X
	Adverse event recording	X	X	X	X	X	X	X
Drug dispenser	Treatment administration		X					
	Adverse event recording		X^c^					

^a^D-8, 8 days (D) before treatment; D0, day of first treatment; D120, D240 and D365, days post first treatment

^a^To rule out suspected heartworm infection despite a negative result after antigen and modified Knott’s test

^b^Performed within 1 h after administration

All owners and all personnel involved in efficacy and safety assessments were blinded to treatment allocation. Day 0 (D0) was defined as the day of the first treatment.

### Laboratory analyses

Antigen and Knott’s tests were performed at 8 days prior to first treatment (D-8), D0 and at 8, 120, 240 and 365 days post first treatment (D8, D120, D240 and D365, respectively) (Table 2). Testing for circulating *D. immitis* antigens was carried out on-site using an immunochromatographic test (SNAP 4DX Plus®; IDEXX Laboratories, Westbrook, ME, USA). Samples that tested positive were subsequently sent to a laboratory for confirmation by enzyme-linked immunosorbent assay (ELISA) (PetChek®; IDEXX Laboratories). Optical densities (OD) values were measured at 650 nm with an Easy Reader (BioRad Laboratories, Hercules, CA, USA). Testing for circulating mf was performed with the modified Knott’s test, according to Knott [8].

### Clinical investigations

Dogs were examined at the same timepoints as the laboratory analyses (D-8, D0, D8, D30, D120, D240 and D365). Evaluation parameters included body condition score (BCS), assessed using a 5-point scale [9], rectal temperature, pulmonary and cardiac auscultation, thoracic radiography and echocardiography to detect potential signs of heartworm disease (HWD), blood sampling for hematology and biochemistry and assessment for signs of macrocyclic lactone toxicity (including depression, ataxia, mydriasis, salivation and muscle fasciculations). Local reactions at injection sites (pain, swelling) were also recorded.

## Results

Of the 114 dogs recruited, 112 completed the study. Of the two dogs that did not complete the study, one dog was excluded at D8 due to a positive Knott’s test for *D. immitis* circulating mf, and the second dog died during the study as a result of septicemia secondary to trauma. All remaining 112 dogs tested negative for *D. immitis* throughout the study, including at endpoints D245 and D365. Clinical investigations carried out throughout the study did not report any changes in body condition score (in both groups, the most represented scores at each visit were 5 and 6), rectal temperature or cardio-respiratory parameters. No signs of HWD were observed on thoracic radiography and echocardiography. An increase in mean alkaline phosphatase values was observed at D245 and D365 in dogs treated with Guardian™ SR, likely due to two dogs who had already shown high values at D0 (from 955 IU/l at D0 to 3405 IU/l at D365 in one dog and from 1137 IU/l at D0 to 1966 II/l at D365 in the second dog another). No adverse events, including signs of macrocyclic lactone toxicity, were observed during the study. No pain or swelling were noted at the injection sites.

## Discussion

The efficacy of ivermectin against *D. immitis* larvae, and its ability to prevent the development of adult heartworms, was first reported in 1982 [10], and in 1987 this drug was introduced in Europe as an oral monthly heartworm preventive. Currently, a wide range of macrocyclic lactones in various formulations, including oral, topical and long-acting injectable formulations, are registered in Europe for the prevention of CHWD [11].

Despite this broad availability of preventive options, and although a recent survey indicated that over 95% of veterinary practitioners in Italy recommend prophylaxis [12], CHWD continues to be diagnosed throughout the country. The same survey reported that veterinary practitioners in northern Italy continue to diagnose the infection in as many as 20 dogs/year [12].

The present study was carried out between 2017 and 2018. There is no evidence that *D. immitis* populations from the two geographic regions where the study was conducted have changed in any way since then. Recent data show that over 15% of dogs living in the Po River valley have circulating *D. immitis* mf, representing an important reservoir of infection [4]. Also, Vismarra et al. [13] recently demonstrated that *D. immitis* continues to circulate in mosquito populations in several areas of the Po River Valley, indicating persistent endemicity and an ongoing risk of infection in dogs that do not receive appropriate preventives. There is also no evidence of ML resistance in *D. immitis* in Italy. The only reported case of ML resistance involved a dog that was adopted from the USA [14]

In the present study, protection against *D. immitis* was 100% for both products evaluated throughout the entire study period. Although plasma concentrations of the active ingredients were not assessed in all treated dogs, a previous study using experimentally infected dogs treated with the implants demonstrated 100% efficacy for up to 12 months following ivermectin implants administration [15].

Owner compliance with monthly heartworm preventives is known to be suboptimal, and poor adherence to recommended protocols may partly explain the persistence and continued circulation of *D. immitis* in endemic regions [16]. Injectable slow-release formulations offer the distinct advantage of shifting responsibility for prevention from owners to veterinary practitioners. FILAPREV® has the additional benefit of being removable in the event of signs of ML toxicity, an option not available with other currently marketed slow-release injectable formulations.

## Conclusions

In conclusion, a single subcutaneous administration of FILAPREV® containing ivermectin implants at the recommended dosage is safe and effective in preventing HWD caused by *D. immitis* in dogs for at least 8 months, covering the full seasonal period of heartworm risk in hyperendemic regions. No adverse events related to the treatment (macrocyclic lactone toxicity, pain/swelling at the injection sites) were reported during the study.

## Data Availability

Data supporting the main conclusions of this study are included in the manuscript.

## Abbreviations

CHWD: Canine heartworm disease
mf: Microfilariae
MLs: Macrocyclic lactones
MSD: Minimum standard dose

## Footnotes

1 FILAPREV® is authorized by the Italian Ministry of Health, for use in Italy

2 Guardian™ SR is authorized through Mutual Recognition Procedure in Italy, France, Greece, Portugal and Spain
